# Human Induced Pluripotent Stem Cell Models of Frontotemporal Dementia With Tau Pathology

**DOI:** 10.3389/fcell.2021.766773

**Published:** 2021-11-10

**Authors:** Rebekka Kühn, Aayushi Mahajan, Peter Canoll, Gunnar Hargus

**Affiliations:** ^1^Department of Pathology and Cell Biology, Columbia University, New York, NY, United States; ^2^Taub Institute for Research on Alzheimer’s Disease and the Aging Brain, Columbia University, New York, NY, United States

**Keywords:** frontotemporal dementia (FTD), induced pluriopotent stem cells, tau, disease modeling, frontotemporal lobar degeneration (FTLD), neurodegenenerative diseases, tauopathy, neurodegeneration

## Abstract

Neurodegenerative dementias are the most common group of neurodegenerative diseases affecting more than 40 million people worldwide. One of these diseases is frontotemporal dementia (FTD), an early onset dementia and one of the leading causes of dementia in people under the age of 60. FTD is a heterogeneous group of neurodegenerative disorders with pathological accumulation of particular proteins in neurons and glial cells including the microtubule-associated protein tau, which is deposited in its hyperphosphorylated form in about half of all patients with FTD. As for other patients with dementia, there is currently no cure for patients with FTD and thus several lines of research focus on the characterization of underlying pathogenic mechanisms with the goal to identify therapeutic targets. In this review, we provide an overview of reported disease phenotypes in induced pluripotent stem cell (iPSC)-derived neurons and glial cells from patients with tau-associated FTD with the aim to highlight recent progress in this fast-moving field of iPSC disease modeling. We put a particular focus on genetic forms of the disease that are linked to mutations in the gene encoding tau and summarize mutation-associated changes in FTD patient cells related to tau splicing and tau phosphorylation, microtubule function and cell metabolism as well as calcium homeostasis and cellular stress. In addition, we discuss challenges and limitations but also opportunities using differentiated patient-derived iPSCs for disease modeling and biomedical research on neurodegenerative diseases including FTD.

## Introduction

**FIGURE 1:**
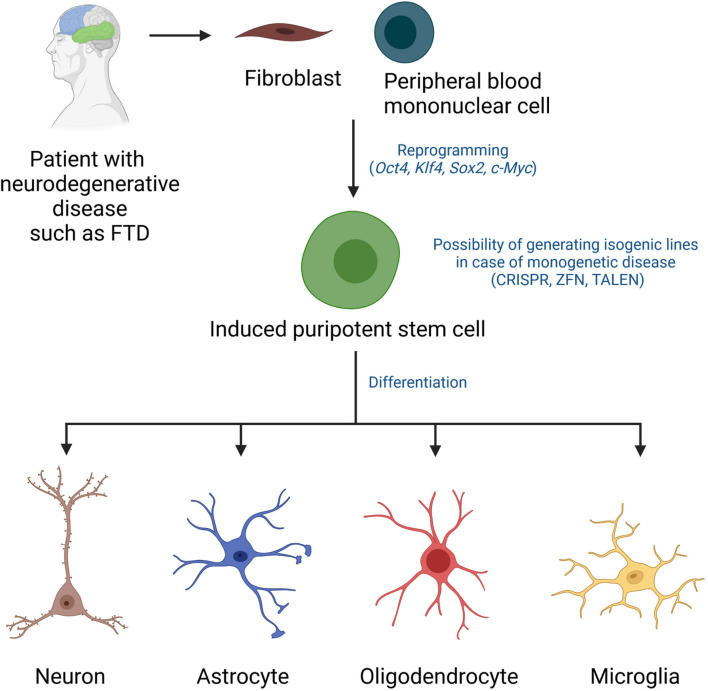
Disease modeling using patient-derived induced pluripotent stem cells. iPSCs can be derived from fibroblasts or peripheral blood mononuclear cells of patients using established reprogramming technologies involving overexpression of the transcription factors *Oct4*, *Klf4*, *Sox2*, *c-Myc*. Gene editing has been applied in various studies to generate isogenic lines. iPSCs from patients and control cells (isogenic iPSCs or healthy donor-derived iPSCs) can be guided to differentiate into neurons, astrocytes, oligodendrocytes, and microglia using optimized differentiation protocols and they can then be tested in various disease modeling and/or drug screening assays. CRISPR, clustered regularly interspaced short palindromic repeats; ZFN, zinc finger nucleases; TALEN, transcription activator-like effector nucleases.

Human induced pluripotent stem cells (iPSCs) have been widely used for research on neurological disorders including neurodegenerative diseases. iPSCs are generated from differentiated somatic cells, usually fibroblasts or peripheral blood mononuclear cells, by overexpression of the reprogramming factors *Oct4*, *Klf4*, *Sox2* and *c-Myc* (Takahashi and Yamanaka, 2006; Takahashi et al., 2007; Figure 1). They share the main characteristics of embryonic stem cells (ESCs) such as the ability for unlimited self-renewal and the potential to differentiate into cells of all three germ layers: ectoderm, mesoderm, and endoderm (Takahashi and Yamanaka, 2006; Takahashi et al., 2007). Thus, iPSCs provide powerful resources to study the differentiation of pluripotent cells into specialized cells of interest, such as functional neurons (Chambers et al., 2009; Shi et al., 2012; Reinhardt et al., 2013a), astrocytes (Serio et al., 2013; Hallmann et al., 2017; Guttikonda et al., 2021), oligodendrocytes (Stacpoole et al., 2013; Ehrlich et al., 2017) or microglia (Muffat et al., 2016; McQuade et al., 2018; Haenseler and Rajendran, 2019).

Induced pluripotent stem cells also represent a unique cell source for high throughput toxicology, vulnerability and drug screens since high numbers of specialized cells can be readily generated from iPSCs, while the isolation of certain living human cells *ex vivo* (e.g., neurons or glial cells from the human brain) is difficult to accomplish due to technical limitations, lack of tissue availability and ethical concerns. Notably, since iPSCs can be derived from biopsies or blood samples of patients, they serve as unique tools to model patient-specific disease processes *in vitro* with the goal to identify mechanisms of disease development, to identify therapeutic targets and to develop customized therapies (Hargus et al., 2014). As such, iPSC models have been established to study pathology in various neurodegenerative disorders such as Alzheimer’s disease (AD) (Penney et al., 2020), Parkinson’s disease (Soldner et al., 2009; Hargus et al., 2010; Reinhardt et al., 2013b), Huntington’s disease (Csobonyeiova et al., 2020) or frontotemporal dementia (FTD) (Hargus et al., 2014; Lines et al., 2020).

Frontotemporal dementia represents 5–15% of all cases with dementia and is the second most common group of early onset dementia after AD with an incidence of 2.7–4.1 cases per 100,000 people annually (Knopman and Roberts, 2011; Rademakers et al., 2012). Patients with FTD typically present with progressive degeneration of the frontal and anterior temporal lobes but additional anatomical structures such as the basal ganglia and several brain stem areas are also affected to various degrees. As a consequence, the presentation of FTD as a clinical syndrome is heterogenous and different clinical variants of FTD exist (Neary et al., 1998; McKhann et al., 2001). Patients with the behavioral variant of FTD (bvFTD) present with changes in personality and executive dysfunction including disinhibition, impulsivity, apathy, loss of empathy, repetitive behaviors and dietary changes. A subset of FTD patients is diagnosed with primary progressive aphasia (PPA), a language abnormality which is associated with difficulties in the retrieval of words (semantic variant of PPA; svPPA) or with an impairment of language production (non-fluent variant of PPA; nfvPPA). Oftentimes, features of bvFTD and PPA overlap with disease progression. FTD is also often paralleled by movement abnormalities such as progressive supranuclear palsy (PSP), corticobasal syndrome (CBS), and/or amyotrophic lateral sclerosis (ALS; FTD-ALS) (Olney et al., 2017).

**FIGURE 2:**
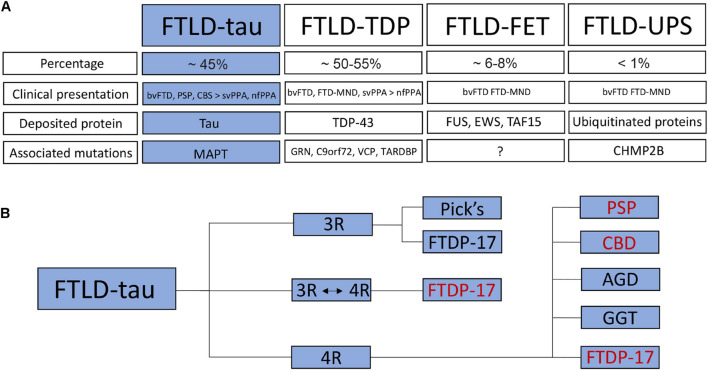
Classification of frontotemporal dementia / frontotemporal lobar degeneration. **(A)** FTLD is subdivided into FTLD-tau with deposition of hyperphosphorylated tau, FTLD-TDP with TDP-43 pathology, FTLD-FET with deposition of FET proteins as well as FTLD-UPS with deposition of ubiquitinated proteins. The percentage, clinical presentation, deposited proteins and associated mutations are listed for each FTLD subtype. **(B)** FTLD-tau (highlighted in blue) is further subdivided into 3R and 4R tauopathies as well as a small group of patients with unaltered 3R:4R ratio. Human iPSC models have been established for some FTLD-tau subtypes (in red). bvFTD, behavioral variant of frontotemporal dementia; PSP, progressive supranuclear palsy; CBS, corticobasal syndrome; svPPA, semantic variant of primary progressive aphasia; nfPPA, non-fluent variant of PPA; FTD-MND, FTD with motor neuron disease; TDP-43, transactive response DNA binding protein molecular weight 43; FUS, fused in sarcoma; EWS, Ewing‘s sarcoma; TAF15, TATA-binding protein-associated factor 15; GRN, granulin; C9orf72, chromosome 9 open reading frame 72; VCP, valosin-containing protein; TARDBP, TAR DNA-binding protein 43; CHMP2B, charged multivesicular body protein 2B; Pick’s, Pick’s disease; FTDP-17, Frontotemporal Dementia and Parkinsonism linked to chromosome 17; CBD, corticobasal degeneration; AGD, argyrophilic grain disease; GGT, globular glial tauopathy.

Due to the involvement of the frontal and temporal lobes, FTD is also referred to as frontotemporal lobar degeneration (FTLD) (Figure 2). Autopsy studies have revealed mislocalization and pathological accumulation of particular intracellular proteins in the brains of patients. These proteins include the microtubule-associated protein tau, the transactive response DNA binding protein molecular weight 43 (TDP-43), fused in sarcoma (FUS), Ewing’s sarcoma (EWS) or TATA-binding protein-associated factor 15 (TAF15) (Rademakers et al., 2012; Mackenzie and Neumann, 2016). These findings led to further subclassification of FTLD into FTLD-tau with deposition of hyperphosphorylated tau (∼45% of all FTLD patients), FTLD-TDP with deposition of TDP-43 (∼50–55% of all FTLD patients) and FTLD-FET with deposition of FET proteins (FUS, EWS, and TAF15; ∼6–8% of all FTLD patients) (Figure 2). A small minority of cases (below 1%) is grouped as FTLD-UPS with deposition of ubiquitinated proteins negative for TDP-43, tau and FET proteins (Figure 2A).

About 20–50% of patients with FTLD have a positive family history and autosomal-dominant patterns of inheritance have been linked to mutations in the following genes/loci associated with the following FTLD subtypes. These genes/loci are *MAPT* encoding tau causing FTLD-tau, *GRN* encoding progranulin causing FTLD-TDP, *C9orf72* (*chromosome 9 open reading frame 72*) causing FTLD-TDP with or without motor neuron disease and representing the most common genetic alteration in ALS, *VCP* encoding the valosin-containing protein causing FTLD-TDP, and *CHMP2B* encoding the charged multivesicular body protein 2B causing FTLD-UPS. Rare cases of FTLD-TDP have been linked to a mutation in *TARDBP* encoding TDP-43 (Figure 2A), although these mutations are usually associated with ALS (Mackenzie and Neumann, 2016).

There are currently no effective disease-modifying therapies for patients with FTD and thus, research efforts aim at identifying mechanisms of pathogenesis and developing therapeutic strategies. Studies on postmortem brain tissue of patients have gained very important insights into the underlying pathology providing information on the involvement of the different brain areas and cell types as well as the deposition of aforementioned disease-associated pathological proteins. However, access to patient brain tissue is limited and postmortem brain studies capture a ‘snapshot’ of the final stage of the disease while data on early molecular events and an understanding of the development of the disease over time are difficult to extract. As an alternative, mouse models of FTD have been developed and could recapitulate some of the pathologic findings seen in patients’ brains (Lewis et al., 2000; Allen et al., 2002; Dawson et al., 2007; Roberson, 2012). However, important biological functions such as splicing of endogenous tau into 3R and 4R tau isoforms, a major dysregulated process in many patients with FTLD-tau as described below, cannot be modeled properly in adult mice since they express only 4R tau endogenous isoforms, limiting proper mechanistic studies and functional assays. Differences in biological processes between humans and mice like these may help to explain why drug trials that had shown promising results in studies on neurodegenerative diseases in mice then failed in human trials. In this context, patient-derived iPSCs may provide powerful tools.

In this review, we will provide an overview of human iPSC models of FTLD-tau. FTLD-tau forms with AD the large group of neurodegenerative tauopathies that are characterized by abundant deposition of hyperphosphorylated tau (p-tau) protein in various brain areas. While both AD and FTLD-tau show pronounced tau pathology, there are distinct differences in p-tau deposition between AD and FTLD-tau in regards to affected neuronal and glial cell types and in regards to the spatio-temporal distribution of p-tau aggregates in the brains. Also, in contrast to AD, the formation of β-amyloid-positive plaques is not a diagnostic feature of FTLD-tau. According to the most recent classification of FTLD, FTLD-tau includes non-AD tauopathies such as Pick’s disease (PiD), PSP, corticobasal degeneration (CBD), argyrophilic grain disease (AGD), globular glial tauopathy (GGT) as well as FTLD-tau linked to mutations in *MAPT* (Mackenzie and Neumann, 2016; Figure 2B).

**FIGURE 3:**
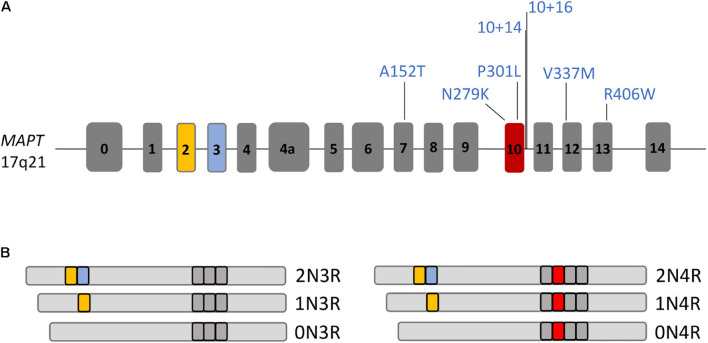
The *MAPT* gene and its different splice isoforms. **(A)** Drawing of the *MAPT* gene composed of 16 exons. *MAPT* mutations, for which human iPSC models with disease phenotypes have been described, are highlighted. **(B)** *MAPT* is spliced into 6 different isoforms in the adult human brain, 0N3R, 1N3R, 2N3R, 0N4R, 1N4R, or 2N4R tau, with presence or absence of exons 2, 3, and 10 in transcripts.

Tau is a cytoplasmic protein that is mainly found in neurons but at lower levels also in glial cells such as astrocytes and oligodendrocytes. Tau is predominantly expressed in the axons of neurons where it binds to microtubules and controls microtubule polymerization and stabilization (Witman et al., 1976; Drechsel et al., 1992). Tau is essential for microtubule-associated functions such as axoplasmic transport of cargo molecules and organelles, axonal outgrowth, dendritic positioning and neuronal polarity (Kanaan et al., 2011; Noble et al., 2013). In addition, tau protects DNA from heat damage and oxidative stress and regulates neuronal excitability (Noble et al., 2013; Sohn et al., 2019). Tau is encoded by the *MAPT* gene on chromosome 17q21 that is composed of 16 exons (Figure 3). Alternative splicing of *MAPT* leads to the formation of six different tau isoforms that contain either 3 or 4 C-terminal microtubule-binding domains depending on alternative splicing of exon 10, which encodes one of these four microtubule-binding domains (3R and 4R tau isoforms) (Goedert et al., 1989). Additional splicing of exons 2 and 3 leads to the formation of three different 3R and three different 4R tau isoforms with either 0 (0N), 1 (1N), or 2 (2N) N-terminal repeats resulting in 0N3R, 1N3R, 2N3R, 0N4R, 1N4R, and 2N4R tau isoforms (Figure 3). In the healthy adult human brain, these 3R and 4R isoforms appear at equal levels (Kosik et al., 1989; Goedert and Jakes, 1990) but in many patients with FTLD-tau including PSP, CBD, AGD, and GGT and including many patients with *MAPT* mutations, this ratio is shifted toward 4R isoforms (Goedert and Spillantini, 2011; Ghetti et al., 2015; Rosler et al., 2019; Figure 2B). Patients with PiD and a few patients with *MAPT* mutations present with a decreased 4R:3R ratio, while no changes in the 4R:3R ratio are noted in some patients with FLTD-tau linked to mutant *MAPT* (Ghetti et al., 2015).

The formation of tau isoforms is developmentally regulated such that the fetal 0N3R isoform appears earliest during development, while the 2N4R tau isoform is the latest to be formed, resulting in the production of all six tau isoforms a few weeks after birth (Goedert et al., 1989). This developmental regulation of tau splicing is also reflected in differentiating human ESCs (Iovino et al., 2010; Sposito et al., 2015) and human iPSCs (Iovino et al., 2015; Sposito et al., 2015). Here, the fetal 0N3R tau isoform appears earliest during differentiation while other tau isoforms are absent. Further maturation over time results in the formation of first the 0N4R isoform with expression of all six isoforms in human iPSC-derived neurons after 150 days (Iovino et al., 2015) and 365 days (Sposito et al., 2015), respectively, of maturation *in vitro*. In addition, it was shown that tau protein is also secreted into the medium supernatant of human iPSC-derived neurons (Esteras et al., 2021). These findings highlight that human iPSCs are suitable for modeling FTLD-tau with the caveat that prolonged time windows of differentiation might be necessary to capture certain disease phenotypes in patient-derived neurons.

## Human Induced Pluripotent Stem Cell Models of Frontotemporal Lobar Degeneration with Tau Pathology

**FIGURE 4:**
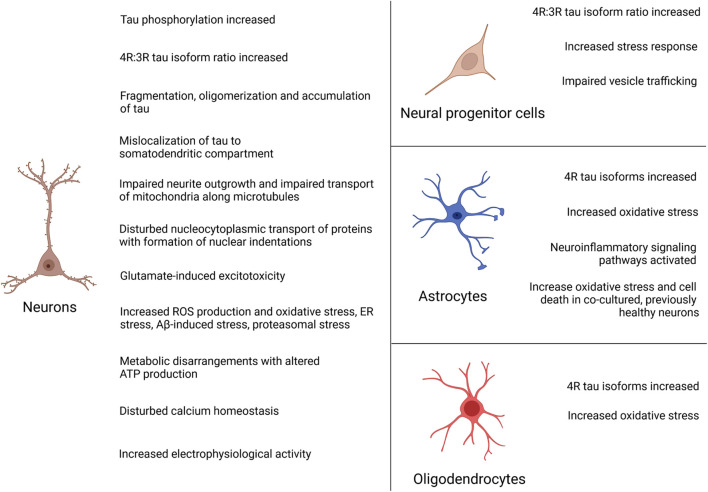
Summary of disease phenotypes in FTLD-tau iPSC-derived neural cells. Disease phenotypes have mainly been described in patient iPSC-derived neurons. In addition, pathologic changes have been reported in neural progenitor cells, astrocytes and oligodendrocytes.

The vast majority of published studies on iPSC models of FTLD-tau is based on reprogrammed fibroblasts from patients with autosomal-dominant mutations in *MAPT* (Tables 1–4). *MAPT* mutations were first described in 1998 in affected individuals of 9 families with FTD with genetic linkage to chromosome 17q21 (Hutton et al., 1998; Poorkaj et al., 1998; Spillantini et al., 1998). *MAPT* mutations are autosomal-dominant, usually fully penetrant and represent about 20% of all mutations in familial FTLD (Moore et al., 2020). Male and females are equally affected (Moore et al., 2020). Sixty seven different *MAPT* mutations have been described so far (Moore et al., 2020) causing early onset FTD and Parkinsonian symptoms in many patients due to the involvement of the substantia nigra and functionally related structures, which resulted in the term Frontotemporal Dementia and Parkinsonism linked to chromosome 17 or FTDP-17 (Ghetti et al., 2015). Patients with *MAPT* mutations form the youngest group of FTD, with a mean age of symptom onset of 49.5 years and a mean disease duration of 9.3 years resulting in a mean age of death at 58.5 years (Moore et al., 2020). The majority of *MAPT* mutations are located in exons 9–13 and in the intron following exon 10 (Clavaguera et al., 2013). Among those, the most common mutations are the *P301L*, *N279K* and the *IVS10 + 16C > T* (henceforth termed 10 + 16) mutations that account for up to 60% of all FTDP-17 cases (Arendt et al., 2016; Moore et al., 2020). iPSC models of FTLD-tau revealed phenotypes linked to, besides others, tau pathology, microtubule function, cell metabolism, calcium homeostasis and dysregulated stress pathways. In the following chapters, we review pathologic changes in differentiated FTLD-tau patient iPSCs grouped by genetic background. A summary of disease phenotypes in different patient-derived neural cell types is provided in Figure 4.

**TABLE 1:** Induced pluripotent stem cell models of FTLD-tau linked to *MAPT* mutations in exon 10.

*MAPT* mutation	Location of mutation / Tau isoforms in brains	iPSC-derived cell type analyzed	Tau pathology in iPSC-derived neural cells	Other disease phenotypes in iPSC-derived neural cells	Isogenic lines (method)	References
N279K	Exon 10 4R tau	Cortical neurons	Increased 4R:3R tau ratio with accelerated and increased expression of 4R tau; increased number of p-tau^+^ neurons; AT100^+^ dot-like staining in few neurons	Accelerated neuronal maturation, reduced anterograde transport of mitochondria	N	Iovino et al., 2015
N279K		Mixed neurons	Increased 4R:3R tau ratio with elevated 4R tau; increased fragmentation of tau; increased number of p-tau+ neurons	Impaired neurite outgrowth; increased ER and oxidative stress; reversal of oxidative stress by coenzyme Q10 and GSK-beta inhibitor; identification of MAGEH1 as neuroprotective factor	N	Ehrlich et al., 2015
N279K		Neural progenitor cells	Increased 4R:3R tau ratio with increased expression of 4R tau and reduced expression of 3R tau	Impaired differentiation of neural progenitor cells into mature neurons; elevated cellular stress with accumulation of stress granules; impaired vesicle trafficking	N	Wren et al., 2015
N279K		Astrocytes	Elevated expression of 4R tau	Increased cell size; increased rotenone-induced oxidative stress; astrocytes increase oxidative stress and cell death in co-cultured healthy neurons	Y (CRISPR)	Hallmann et al., 2017
N279K		Oligo-dendrocytes	Aberrant expression of 4R tau	Increased rotenone-induced oxidative stress	Y (CRISPR)	Ehrlich et al., 2017

P301L	Exon 10 4R tau	Cortical neurons	Increased number of p-tau^+^ neurons; formation of contorted neuronal processes with varicosities containing alpha-synuclein and 4R tau while 4R:3R tau ratio not altered	Accelerated neuronal maturation; reduced anterograde and retrograde transport of mitochondria	N	Iovino et al., 2015
P301L		Cerebral organoids	Generation of P301L;Δp35KI results in reduced tau phosphorylation and increased expression of synaptophysin	Increased p25/p35 ratio	N/Y (CRISPR)	Seo et al., 2017
P301L		Mixed neurons	Tau-degrading agent QC-01–175, a PET tracer derivative, reduces total tau and p-tau and preferentially degrades tau species in *MAPT*-mutant neurons	Increased vulnerability towards Aβ-induced cell stress	N	Silva et al., 2019
P301L		Cortical neurons	Increased phosphorylation and mislocalization of tau to cell body and dendrites	Indentation and deformation of nuclear envelope with disrupted nucleocytoplasmic transport; microtubule depolymerization with nocodazole restores nuclear morphology and nucleocytoplasmic transport	Y (TALEN)	Paonessa et al., 2019
P301L		iPSCs	N/A	iPSCs as resource	Y/N	Karch et al., 2019

S305I S305N S305S	Exon 10 4R tau	iPSCs	N/A	iPSCs as resource	Y N Y	Nimsanor et al., 2016a; Karch et al., 2019

**TABLE 2:** Induced pluripotent stem cell models of FTLD-tau linked to *MAPT* mutations in intron 10.

*MAPT* mutation	Location of mutation / Tau isoforms in brains	iPSC-derived cell type analyzed	Tau pathology in iPSC-derived neural cells	Other disease phenotypes in iPSC-derived neural cells	Isogenic lines (method)	References
10 + 14 C > T	Intron 10 4R tau	Cortical neurons	Increased 4R:3R tau ratio with increased expression of 4R tau; accumulation of intracellular misfolded tau with formation of intracellular puncta and dots; release of misfolded tau protein into medium supernatant	Increased calcium levels after electrical stimulation; increased spontaneous cell death; inhibition of calcium influx via the AMPA receptor inhibitor CNQX or the NMDA receptor inhibitor AP-5 increases cell survival	Y (CRISPR)	Imamura et al., 2016

10 + 16 C > T	Intron 10 4R tau	Cortical neurons	Increased 4R:3R tau ratio with accelerated and increased expression of 4R tau during neuronal differentiation of 10 + 16 neurons	N/A	N	Sposito et al., 2015
10 + 16 C > T		Cortical neurons	Increased 4R:3R tau ratio	Increased neuronal vulnerability under baseline culture conditions and after rapamycin-induced cell stress; increased levels of MMP-9 and MMP-2; inhibition of MMP-9/MMP-2 protects cells from death	N	Biswas et al., 2016
10 + 16 C > T		Cortical neurons	Accelerated expression of 4R tau in 10 + 16 neurons after transplantation into the frontal cortex of newborn APP PS1 tg/wt *Prkdc*^scid/scid^ mice	N/A	N	Espuny-Camacho et al., 2017
10 + 16 C > T		Cortical neurons	N/A	Increased mitochondrial membrane potential; increased ROS and oxidative stress; lower mitochondrial NADH pool with decreased mitochondrial respiration / oxidative phosphorylation and increased glycolysis; altered mechanisms of ATP production; partial prevention of cell death through application of antioxidant MitoQ	N	Esteras et al., 2017
10 + 16 C > T		Cortical neurons	Increased 4R:3R tau ratio with accelerated and increased expression of 4R tau; increased expression of p-tau	Differences in neuronal subtype specification with reduction of glutamatergic and upregulation of GABAergic markers; reduced proliferation of neural progenitor cells; aberrant WNT and SHH signaling	Y (ZFN)	Verheyen et al., 2018
10 + 16 C > T		Cortical neurons	Increased phosphorylation and mislocalization of tau to cell body and dendrites	Indentation and deformation of nuclear envelope with disrupted nucleocytoplasmic transport; nocodazole restores nuclear morphology and nucleocytoplasmic transport; confirmation of altered nuclear morphology in postmortem brain tissue of patients	N	Paonessa et al., 2019
10 + 16 C > T		Cortical neurons	N/A	Exogenous 4R increases spontaneous calcium oscillations; glutamate induces increased calcium influx and mitochondrial depolarization; mitochondrial calcium overload induced by ferutinin leads to accelerated cell death	N	Britti et al., 2020
10 + 16 C > T		Cortical neurons	N/A	Increased membrane excitability; functional downregulation of voltage-gated Na^+^ and K^+^ channels and reduced expression of Nav1.6; altered characteristics of action potentials	N	Kopach et al., 2020
10 + 16 C > T		Cortical neurons	N/A	Impaired excitability with depolarized resting membrane potential and increased input resistance; decreased voltage-gated Na^+^ and K^+^ currents; reduced expression of Nav1.6; impaired ability to fire action potentials with altered AP waveform; suppressed intracellular Ca^++^ dynamics in dendrites and soma during depolarization	Y (ZFN)	Kopach et al., 2021

10 + 16 C > T	Intron 10 4R tau	Cortical neurons	N/A	Overproduction of ROS; increased expression of AMPA and NMDA receptors containing GluA1 and NR2B subunits leading to altered glutamatergic signaling, calcium overload and excitotoxicity; application of antioxidants MitoQ and MitoTEMPO prevents cell death; conditioned medium from 10 + 16 neurons or exogenous 4R tau (K18 fragment) alters glutamate-induced Ca^++^response in healthy neurons by increasing mitochondrial ROS production; 4R tau induces excitotoxicity and cell death; rescue by MitoQ and glutamate receptor antagonist CNQX	Y/N (ZFN)	Esteras et al., 2021
10 + 16 C > T		iPSCs	N/A	iPSCs as resource	Y/N	Karch et al., 2019

10 + 16 C > T/P301S	Intron 10 Exon 10 4R tau	Cortical neurons	Increased 4R:3R tau ratio with accelerated and increased expression of 4R tau; introduction of the P301S mutation in 10 + 16 iPSCs promotes tau oligomerization	Introduction of the P301S mutation in 10 + 16 iPSCs increases calcium burst frequency and reduces lysosomal acidity	Y (ZFN)	Verheyen et al., 2018

10 + 16 C > T/P301L/N279K	Intron 10 Exon 10 4R tau	Cortical neurons	Increased 4R:3R tau ratio with accelerated and increased expression of 4R tau during neuronal differentiation; increased phosphorylation and mislocalization of tau to cell body; tau aggregation after overexpression of P301L *MAPT* and in the presence of exogenous 4R tau (K18 fragment)	Increased electrophysiological activity; altered and accelerated cortical differentiation with reduced glutamatergic and increased GABAergic marker gene expression; altered neurite outgrowth; increased oxidative, endoplasmic reticulum and inflammatory stress with increased cell death	Y (CRISPR)	Garcia-Leon et al., 2018

**TABLE 3:** Induced pluripotent stem cell models of FTLD-tau linked to *MAPT* mutations in exons 12 and 13.

*MAPT* mutation	Location of mutation / Tau isoforms in brains	iPSC-derived cell type analyzed	Tau pathology in iPSC-derived neural cells	Other disease phenotypes in iPSC-derived neural cells	Isogenic lines (method)	References
V337M	Exon 12 3R and 4R tau	Mixed neurons	Increased fragmentation of tau; increased number of p-tau^+^ neurons; 4R:3R tau ratio unaltered	Impaired neurite outgrowth; increased ER and oxidative stress; reversal of oxidative stress by coenzyme Q10 and GSK-beta inhibitor; identification of MAGEH1 as neuroprotective factor	N	Ehrlich et al., 2015
V337M		Cortical neurons	N/A	Impaired activity-dependent plasticity of cytoskeleton in the AIS by accumulation of EB3; impaired homeostatic control of neuronal excitability with increased firing rate; gene correction and reduction of EB3 levels via siRNA restore AIS plasticity	Y (CRISPR)	Sohn et al., 2019
V337M		Cerebral organoids	Progressive accumulation of total tau and p-tau	Accelerated differentiation and reduced survival of cortical glutamatergic neurons; dysfunction of the early autophagy-lysosomal pathway; increased formation of stress granules; dysregulation of gene splicing; increased expression of ELAVL4; increased susceptibility to glutamate-induced excitotoxicity with rescue by glutamate receptor inhibitors and by the PIKFYVE kinase inhibitor apilimod; upregulation of neuroinflammation signaling pathways in astrocytes	Y (CRISPR)	Bowles et al., 2021
V337M		iPSCs	N/A	iPSCs as resource	Y/N	Karch et al., 2019

G389R	Exon 13 3R and 4R tau	iPSCs	N/A	iPSCs as resource	N	Karch et al., 2019

R406W	Exon 13 3R and 4R tau	Cortical neurons	Accumulation of intracellular misfolded tau with formation of puncta and dots; release of misfolded tau protein into medium supernatant; increased calcium levels after electrical stimulation	Increased spontaneous cell death; inhibition of calcium influx via the AMPA receptor inhibitor CNQX or the NMDA receptor inhibitor AP-5 increases cell survival	N	Imamura et al., 2016
R406W		Cortical neurons	N/A	Characterization of transcriptome profiles in *MAPT* R406W iPSC-derived neurons and in postmortem brain tissue from patients with *MAPT* R406W. Identification of 61 overlapping genes linked to calcium-dependent presynaptic function and GABAergic signaling; genes significantly enriched for FTD risk variants; some overlap of gene expression signatures in *MAPT* R406W iPSC-derived neurons with also postmortem brain tissue of PSP patients	Y (CRISPR)	Jiang et al., 2018
R406W		Cerebral organoids/cortical neurons	Reduced phosphorylation of tau protein in patient neurons; increased fragmentation of tau protein with an increased cleavage by calpain; mislocalization of tau within neurons	Formation of βIII-tubulin^+^ puncta and impaired mitochondrial transport with rescue by the microtubule-stabilizing compound Epothilone D	Y (CRISPR)	Nakamura et al., 2019
R406W		iPSCs	N/A	iPSCs as resource	Y/N (CRISPR)	Nimsanor et al., 2016b, c; Rasmussen et al., 2016a, b; Karch et al., 2019

**TABLE 4:** Additional induced pluripotent stem cell models of FTLD-tau.

*MAPT* variant	Location of variant / Tau isoforms in brains	iPSC-derived cell type analyzed	Tau pathology in iPSC-derived neural cells	Other disease phenotypes in iPSC-derived neural cells	Isogenic lines (method)	References
A152T	Exon 7 4R tau	Mixed neurons	Shortening, bending and fragmentation of neurites; mislocalization of tau to the somatodendritic compartment; increased p-tau expression and increased numbers of p-tau-positive neurons; increased fragmentation of tau; aggravation of phenotypes in neurons homozygous for the mutation	Increased vulnerability of dopaminergic neurons	Y (ZFN)	Fong et al., 2013
A152T		Cortical neurons	Increased 4R:3R tau ratio	Increased neuronal vulnerability under baseline culture conditions and after rapamycin-induced cell stress; increased levels of MMP-9 and MMP-2; inhibition of MMP-9/MMP-2 protects against cell death; MMP-9 activation is dependent on ERK phosphorylation	N	Biswas et al., 2016
A152T		Cortical neurons	Accelerated and increased expression of tau and p-tau; accumulation but no aggregate formation; somatodendritic redistribution of p-tau; increased posttranslational modification of tau by mass spectroscopy; reduced solubility of tau; 4R:3R tau isoform ratio unaltered	Activated autophagy; increased protein ubiquitination and ER stress; increased vulnerability towards oxidative stress, excitotoxicity, proteasomal stress and Aβ-(1–42); rescue by reducing/disrupting expression of tau	N	Silva et al., 2016
A152T		Mixed neurons	Tau-degrading agent QC-01–175 reduces total tau and p-tau; preferentially degrades tau species in *MAPT*-mutant neurons	Increased vulnerability towards Aβ-induced cell stress and rescue by QC-01-175	N	Silva et al., 2019
A152T		Cortical neurons	Increased expression of tau and p-tau; reduction of p-tau in cell processes and cell body after application of the GSK3 inhibitor CHIR-99021, the kinase inhibitors enzastaurin and ruboxistaurin as well as the small molecule kinase inhibitors AT7519 and CGP-60474; CHIR-99021 and enzastaurin also decrease levels of total tau	N/A	N	Cheng et al., 2021
A152T		iPSCs	N/A	iPSCs as resource including patient with CBD	N	Karch et al., 2019

Sporadic (PSP)	WT 4R tau	iPSCs	N/A	iPSCs as resource from patients with PSP	N	Karch et al., 2019

### Frontotemporal Lobar Degeneration With Tau Pathology Caused by *MAPT*^*N*279*K*^

Patients with the N279K mutation have the lowest age of onset of all familial forms of FTLD with a mean age at onset of 43.8 years (Moore et al., 2020). Tau pathology is seen in both neurons and glial cells. This mutation affects both peptide sequence and alternative splicing of the tau gene with increased production of 4R tau (Hasegawa et al., 1999; Kar et al., 2005).

Cultures composed of iPSC-derived neurons with the N279K mutation demonstrated an increased number of p-tau-positive neurons and an increased 4R:3R tau isoform ratio with an increased and accelerated expression of 4R tau (Iovino et al., 2015). This observation was paralleled by the formation of p-tau-positive dot-like aggregates in a subset of neurons (Iovino et al., 2015). Furthermore, an accelerated neuronal maturation and an impairment of intracellular transport along microtubules with significantly reduced anterograde transport of mitochondria was noted (Iovino et al., 2015).

Pronounced tau pathology in N279K patient iPSC-derived neurons was also observed in an independent study (Ehrlich et al., 2015) where patient neurons exhibited changes in the 4R:3R tau isoform ratio with elevated levels of 4R tau and an increased fragmentation of tau already 28 days after neuronal maturation of iPSC-derived neural progenitor cells (NPCs). These neuronal cultures also contained an increased number of p-tau-positive neurons and demonstrated an impairment of neurite outgrowth indicating disturbed microtubule function. Furthermore, N279K mutant neurons were much more susceptible to oxidative stress induced by rotenone, an inhibitor of complex 1 of the mitochondrial respiratory chain. This stress response could, at least in part, be rescued by the antioxidant coenzyme Q10 and the GSK-3 inhibitor CHIR99021, providing proof of the concept that stress responses are targetable and partially reversible in patient neurons. This study also described increased endoplasmic reticulum (ER) stress in patient iPSC-derived neurons and in postmortem patient brain tissue, and identified the cytoplasmic protein MAGEH1 as a neuroprotective molecule in patient neurons *in vitro* with an increased expression of MAGEH1 also in patient brains.

An increased stress response with an intracellular accumulation of stress granules, increased caspase-3/7 activity and an elevated ratio of 4R tau to 3R tau with an increased 4R tau but reduced 3R tau expression was already observed in iPSC-derived NPCs from patients with N279K *MAPT* (Wren et al., 2015). Interestingly, these N279K NPCs demonstrated an impaired ability to differentiate into neurons and showed disrupted endocytic trafficking with an accumulation and enlargement of exosomes and endosomes positive for Flotillin-1, a lipid raft marker, with a reduction of lysosomes (Wren et al., 2015). These observations were also confirmed in postmortem brain tissue from patients with mutant N279K *MAPT*, which showed increased levels of Flotillin-1 in the frontal and temporal cortex, indicating that abnormal vesicle trafficking is a pathogenic mechanism in FTLD-tau (Wren et al., 2015).

Induced pluripotent stem cells from patients with the N279K mutation were also differentiated into NPCs, at which stage CRISPR/Cas9 genome editing was performed to generate isogenic control cells (Hallmann et al., 2017). These patient and isogenic control cells were then differentiated into astrocytes by overexpression of *Sox10* and application of pro-astrocytic growth and neurotrophic factors including ciliary neurotrophic factor (Hallmann et al., 2017). In the brains of many patients with mutant *MAPT* including N279K, astrocytes show pronounced tau pathology (Ghetti et al., 2015). Here, tufted astrocytes (Ghetti et al., 2015; Kovacs, 2020) are formed that demonstrate increased expression of 4R tau and abundant deposition of p-tau in the proximal segments of astrocytic processes. Both N279K patient and control iPSC-derived NPCs differentiated into astrocytes at comparable efficiencies and exhibited typical astrocyte-related properties including an uptake of glutamate from the cell culture medium and propagation of calcium waves across gap junctions (Hallmann et al., 2017). However, patient astrocytes appeared significantly larger, showed an aberrant expression of 4R tau and were much more vulnerable to oxidative stress compared to gene-corrected control cells (Hallmann et al., 2017). Interestingly, concentrations of rotenone that were needed to induce cell death in patient iPSC-derived astrocytes were 10 times higher than those needed to induce death in patient iPSC-derived neurons, reflecting differential vulnerabilities of these neural cells in the human brain. This iPSC-based study also demonstrated astrocyte-mediated non-cell autonomous mechanisms of neuronal degeneration in FTLD-tau. This was highlighted by co-culture of either patient or control astrocytes with healthy iPSC-derived control neurons that consequently acquired an increased susceptibility to oxidative stress in the presence of patient but not control astrocytes. These increased stress responses in previously healthy neurons were paralleled by an enrichment of stress-associated pathways in whole transcriptome analyses, further validating observed findings of cell non-autonomy (Hallmann et al., 2017).

In addition to neurons and astrocytes, oligodendrocytes degenerate in the brains of many patients with FTLD-tau linked to mutant *MAPT* (Ghetti et al., 2015). Hyperphosphorylated tau is deposited as coiled bodies in oligodendrocytes, most likely contributing to disease pathogenesis. Oligodendrocytes have been differentiated from human iPSCs through an NPC intermediate by overexpression of the transcription factors *Sox10*, *Olig2*, and *Nkx6.2* and by the application of pro-glial factors and small molecules such as triiodo-L-thyronine (T3), platelet-derived growth factor (PDGF) and smoothened agonist (SAG) (Ehrlich et al., 2017). These oligodendrocytes had the ability to myelinate axons *in vitro* and to form myelin sheaths 16 weeks after the transplantation into the corpus callosum of newborn myelin basic protein (MBP)-deficient shiverer mice that lack the ability to produce myelin (Ehrlich et al., 2017). In this study, oligodendrocytes were also differentiated from N279K patient NPCs along with their isogenic control cells. These cells were not transplanted into shiverer mice but they showed pathologic changes *in vitro* with aberrant expression of 4R tau in differentiated patient oligodendrocytes. Furthermore, N279K patient oligodendrocytes were more susceptible to rotenone-induced oxidative stress with an increased expression of cleaved caspase-3 (Ehrlich et al., 2017).

### Frontotemporal Lobar Degeneration With Tau Pathology Caused by *MAPT*^*P*301*L*^

The P301L *MAPT* mutation is the most common mutation in *MAPT*. This mutation is located in exon 10 and is a missense mutation (Kar et al., 2005) that makes tau more prone to phosphorylation (Alonso Adel et al., 2004). Furthermore, it is predicted to affect binding of the tau protein to microtubules (Kar et al., 2005; Iovino et al., 2015). Patients with the P301L mutation demonstrate abundant deposition of p-tau in neurons, astrocytes and oligodendrocytes, which is paralleled by increased 4R tau isoform expression (Mirra et al., 1999).

In one of the first reports on FTLD-tau iPSCs, stem cells were derived from two patients with the P301L *MAPT* mutation that demonstrated an accelerated neuronal maturation with formation of contorted neuronal processes with varicosities that, at least in a subset, contained alpha-synuclein and 4R tau without affecting the 4R:3R tau ratio (Iovino et al., 2015). Cultures composed of P301L neurons contained an increased number of p-tau-positive neurons and P301L neurons demonstrated a disturbed transport machinery along microtubules with reduced anterograde and retrograde transport of mitochondria (Iovino et al., 2015). Later, it was shown that increased phosphorylation of tau in P301L iPSC-derived neurons was accompanied by p-tau mislocalization to the cell body and dendrites (Paonessa et al., 2019). Such mislocalization of p-tau represents an early pathogenic event in tauopathies (Kowall and Kosik, 1987; Gotz et al., 1995) and was paralleled by indentation and deformation of the nuclear membrane in P301L neurons (Paonessa et al., 2019). These morphological changes were mediated by microtubules and resulted in disrupted protein transport across the nuclear membrane. Importantly, microtubule depolymerization with the small molecule nocodazole reduced these indentations, restored round nuclear morphology and rescued impaired nucleocytoplasmic transport in P301L neurons (Paonessa et al., 2019), further indicating that observed effects were directly mediated by microtubules.

Increased phosphorylation of the tau protein in P301L neural cells was mechanistically linked to p25, a proteolytic subunit of p35 and activator of cyclin-dependent kinase 5 (Cdk5) that, besides tau hyperphosphorylation, has been associated with tau aggregation, accumulation of β-amyloid, neuroinflammation and synaptic loss (Noble et al., 2003; Seo et al., 2017). In this study, P301L patient iPSC-derived cerebral organoids demonstrated increased tau phosphorylation that could be significantly reduced by disrupting of p25/Cdk5 activity via CRISPR/Cas9 editing of the p35 locus (Seo et al., 2017). This approach also resulted in an increased expression of synaptophysin in P301L organoids, overall demonstrating that disrupting p25/Cdk5 activity can ameliorate disease phenotypes in FTLD-tau (Seo et al., 2017). Tau phenotypes in P301L neurons could also be reversed with targeted protein degradation technology to transform the tau positron emission tomography (PET) tracer ^18^F-T807 into the tau degrader QC-01–175 (Silva et al., 2019). This study showed that P301L iPSC-derived neurons were more vulnerable to the aggregation-prone peptide Aβ(1-42) and that QC-01–175 reduced levels of total tau and levels of p-tau in P301L neurons (Silva et al., 2019). Notably, QC-01–175 preferentially degraded tau species in patient-derived neurons, while sparing tau in healthy controls, providing a potential therapeutic approach in neutralizing the neurotoxic effects of tau in tauopathies such as FTLD-tau (Silva et al., 2019).

### Frontotemporal Lobar Degeneration With Tau Pathology Caused by *MAPT*^10 + 16^

Several mutations have been reported in intron 10 of *MAPT* located in the 5′-splice site of the intron following exon 10 (10 + 3, 10 + 11, 10 + 12, 10 + 13, 10 + 14, 10 + 16). These intronic mutations destabilize a stem-loop structure at the exon 10 5′-splice site intron junction resulting in a more frequent usage of the 5′-splice site with altered mRNA splicing of exon 10 and increased levels of 4R tau isoforms (Hutton et al., 1998; Grover et al., 1999).

The *MAPT* 10 + 16 mutation (Janssen et al., 2002; Lantos et al., 2002) is the most common of these intronic mutations and leads to a two- to six-fold increase of exon 10-containing *MAPT* mRNA (Connell et al., 2005). As a result, the brains of patients with the 10 + 16 mutation contain p-tau-positive inclusions in neurons, astrocytes and oligodendroglia that are composed of 4R tau (Ghetti et al., 2015).

In a first report on patient-derived iPSCs carrying the 10 + 16 mutation, expression of 4R tau was significantly accelerated during neuronal maturation resulting in an increased 4R:3R tau isoform ratio in these cells (Sposito et al., 2015). 10 + 16 patient neurons expressed the earliest 4R tau isoforms (0N4R) already at 100 days of differentiation, while only the fetal 3R tau isoform (0N3R) was expressed in cultured control neurons at this time (Sposito et al., 2015). Interestingly, 10 + 16 neurons showed an accelerated expression of 4R tau protein also after transplantation into a mouse model of AD (Espuny-Camacho et al., 2017). While no 4R tau expression was seen 2 months after injection of 10 + 16 NPCs into the frontal cortex of newborn APP PS1 tg/wt *Prkdc*^*scid/scid*^ mice, grafted 10 + 16 neurons expressed 4R tau at 4 months (37% of grafted neurons) and at 6 months (84% of grafted neurons) after cell injection. In contrast, control human ESC-derived neurons did not express 4R tau until 6 months after transplantation into these mice (Espuny-Camacho et al., 2017). These findings demonstrated that accelerated tau splicing in 10 + 16 neurons occurred during similar time frames *in vitro* and *in vivo*. An accelerated expression of 4R tau in *MAPT* 10 + 16 iPSC-derived neurons with an increased 4R:3R tau ratio was confirmed in additional reports (Biswas et al., 2016; Verheyen et al., 2018).

It was also demonstrated that tau is hyperphosphorylated in 10 + 16 iPSC-derived neurons and that p-tau is mislocalized to the cell body and dendrites (Paonessa et al., 2019). The 10 + 16 neurons presented with nuclear indentations and nuclear deformities with disrupted nucleocytoplasmic transport of proteins, as similarly seen in iPSC-derived neurons carrying the P301L mutation. Likewise, these phenotypes were rescued by addition of the microtubule depolymerizing small molecule nocodazole (Paonessa et al., 2019) highlighting that these different *MAPT* mutations can lead to similar reversable morphological and functional nuclear phenotypes. Since the authors had access to postmortem brain tissue from several patients with the 10 + 16 mutation, they examined nuclear morphology in cortical brain samples from the frontal and temporal lobes. These studies identified an increased abundance of nuclei with nuclear lamina invaginations in deep cortical layers in these brain areas. Furthermore, invaginations were especially seen in neurons that contained high levels of p-tau and neurofibrillary tangles, validating the iPSC-model of FTLD-tau linked to *MAPT* 10 + 16 (Paonessa et al., 2019).

Patient-derived 10 + 16 neurons showed decreased survival at baseline culture conditions and demonstrated an increased vulnerability toward rapamycin-induced cell stress, suggesting that the mTOR signaling pathway was functionally altered in these cells (Biswas et al., 2016). Interestingly, increased rapamycin-induced vulnerability was, at least partially, rescued by blockage of the metalloproteinases MMP-9 and MMP-2, which were significantly upregulated and activated in 10 + 16 iPSC-derived neurons and which, by themselves, were able to induce cell death in human iPSC-derived control neurons (Biswas et al., 2016). These findings underscored a detrimental role of MMP-2 and MPP-9 on the survival of neurons, as previously described for MMP-9 for motor neurons in the context of ALS (Kaplan et al., 2014).

Additional studies demonstrated altered mitochondrial function in iPSC-derived neurons from patients with the 10 + 16 mutation (Esteras et al., 2017, 2021). 10 + 16 neurons carried an increased mitochondrial membrane potential leading to overproduction of cytosolic and mitochondrial reactive oxygen species (ROS). This in turn resulted in increased oxidative stress, as indicated by elevated lipid peroxidation, and it led to increased cell death that could be partially prevented by the application of the antioxidant compound MitoQ (Esteras et al., 2017). Interestingly, ATP production was also altered in 10 + 16 neurons. In fact, glycolysis was the main source of ATP in 10 + 16 neurons and ATP production in 10 + 16 neurons was much more dependent on glycolysis as energy fuel when compared to healthy control neurons. On the other hand, 10 + 16 neurons showed lower production of ATP by oxidative phosphorylation, indicating significant disturbances of metabolic programs in these cells (Esteras et al., 2017).

10 + 16 neurons also showed altered electrophysiological properties. Compared to control neurons, 10 + 16 patient-derived neurons demonstrated an increased membrane excitability after about 150 days of differentiation with functional downregulation of voltage-gated Na^+^ and K^+^ channels and reduced expression of the sodium channel Nav1.6, which is known to contribute to the initiation and propagation of action potentials (APs) (Kopach et al., 2020). In line with these observations, recordings revealed altered characteristics of action potentials (AP) in these cells. 10 + 16 neurons exhibited a depolarized AP threshold, a reduced amplitude and altered shape of the induced AP spike and the need for an almost two-fold stronger current that was needed to trigger neurons to fire an AP (Kopach et al., 2020). Furthermore, 10 + 16 patient-derived cortical neurons showed increased expression of AMPA and NMDA receptors containing GluA1 and NR2B subunits leading to altered glutamatergic signaling, calcium overload and excitotoxicity (Esteras et al., 2021). The application of mitochondrial antioxidants MitoQ and MitoTEMPO to the cell culture media led to recovery of impaired Ca^++^ signaling and to prevention of cell death (Esteras et al., 2021) as described for MitoQ before (Esteras et al., 2017).

Interestingly, conditioned medium from patient iPSC-derived 10 + 16 neurons as well as exogenous 4R tau (provided as K18 fragment comprising the four-repeat region of tau) altered glutamate-induced Ca^++^ response when added to cultures of healthy control iPSC-derived neurons (Esteras et al., 2021). This 4R tau treatment led to increased mitochondrial ROS production, induced excitotoxicity and eventually cell death which could be prevented by adding the antioxidant MitoQ or the glutamate AMPA receptor inhibitor CNQX to cultured neurons (Esteras et al., 2021). In addition, when added to patient iPSC-derived 10 + 16 neurons, exogenous 4R tau (K18 fragment) also increased calcium oscillations and glutamate elevated calcium influx with an increased mitochondrial depolarization (Britti et al., 2020). Increased calcium concentrations in patient neurons had detrimental effects, since mitochondrial calcium overload – induced by ferutinin – led to increased vulnerabiltiy and accelerated cell death in patient 10 + 16 neurons when compared to control neurons (Britti et al., 2020). These iPSC-based studies highlight that disturbed calcium homeostasis plays a critical role in the pathogenesis of FTLD-tau-linked *MAPT* 10 + 16.

For different sets of studies, zinc finger nuclease (ZFN) technology was applied to introduce the 10 + 16 mutation in iPSCs from a healthy donor and disease phenotypes were analyzed in genetically engineered neurons in comparison to isogenic controls (Verheyen et al., 2018). 10 + 16 mutant cells were generated as heterozygous and homozygous versions, which showed similar disease phenotypes. Those included reduced proliferation of NPCs, an accelerated expression of 4R tau isoforms during neuronal differentiation with an increased 4R:3R tau ratio, an increased expression of p-tau, aberrant WNT and SHH signaling, and significant differences in neuronal subtype specification with a reduction of glutamatergic markers and an upregulation of GABAergic markers in differentiated cortical neurons. In addition, 10 + 16 neurons showed an upregulation of *TARDBP* encoding the TDP-43 protein and a downregulation of *APOE* and *GRN*. Many of these phenotypes were also observed in neurons, that had been differentiated in parallel and for confirmation from a patient carrying *MAPT* 10 + 16 (Verheyen et al., 2018).

Interestingly, the authors of this paper also introduced a homozygous P301S mutation in homozygous 10 + 16 iPSCs to further aggravate pathologic changes in differentiated neurons (Verheyen et al., 2018). The P301S mutation is a *MAPT* missense mutation in the same locus as the P301L mutation that leads to tau pathology in neurons and glial cells. Tau pathology linked to P301S encompasses an impaired binding of tau to microtubules, a reduced ability of tau to promote microtubule assembly and an increased expression of 4R tau isoforms (Bugiani et al., 1999; Kar et al., 2005). In line with these known observations, the introduction of the P301S mutation in 10 + 16 iPSCs promoted tau oligomerization, but no aggregation, in differentiated neurons in the presence of recombinant human P301L mutant K18 tau fibrils as seeding ground. These changes were not observed in the absence of the P301S mutation and they confirmed a aggregation-promoting function of P301S tau. These findings were paralleled by reduced acidity of lysosomes and increased apoptosis. Furthermore, live cell calcium imaging revealed a significantly increased calcium burst frequency in 10 + 16 neurons carrying the P301S mutation indicating that the P301S mutation induced changes in neuronal excitability, which may contribute to an increased vulnerability of these cells and which may be linked to seizure activity seen in patients with the P301S mutation (Sperfeld et al., 1999; Verheyen et al., 2018).

In follow-up studies, genetically engineered 10 + 16 iPSC lines lacking the P301S mutation were used to further analyze electrophysiological properties in differentiated 10 + 16 neurons in comparison to isogenic controls (Esteras et al., 2021; Kopach et al., 2021). The results of these studies reproduced many findings from patient-derived 10 + 16 neurons and included an impaired excitability with depolarized resting membrane potential and increased input resistance, decreased voltage-gated Na^+^ and K^+^ currents, a reduced expression of Nav1.6, an impaired ability to fire action potentials with altered AP waveforms as well as suppressed intracellular Ca^++^ dynamics in dendrites and soma during depolarization as visualized by multiphoton fluorescent imaging (Kopach et al., 2021). Furthermore, genetically engineered 10 + 16 neurons demonstrated an increased mitochondrial membrane potential and two-photon excitation imaging showed increased glutamate-induced calcium influx (Esteras et al., 2021). These findings were paralleled by increased cytosolic and mitochondrial ROS production as similarly seen in patient iPSC-derived 10 + 16 neurons (Esteras et al., 2017).

In an unrelated study, CRISPR-FokI and piggyBac transposase technology was applied to introduce heterozygous P301L, N279K, and 10 + 16 *MAPT* mutations in healthy control iPSCs (Garcia-Leon et al., 2018). Differentiating triple-*MAPT*-mutant neurons showed an accelerated expression of 4R tau isoforms with an increased 4R:3R tau ratio already at 32 days of differentiation versus 160 days in parental control neurons. The mutant neurons demonstrated increased phosphorylation and mislocalization of tau to the cell body. Interestingly, aggregation of tau was also detected in triple-*MAPT*-mutant neurons when transduced with an adeno-associated viral vector encoding P301L *MAPT*. Such tau aggregation was further enhanced in the presence of exogenous 4R tau (K18 fragment). Triple-*MAPT*-mutant neurons also displayed altered neurite outgrowth, they were electrophysiologically more active when co-cultured on murine primary astrocytes and they showed an altered and accelerated differentiation into cortical neurons with an elevated expression of GABAergic and deep cortical markers and a reduced expression of glutamatergic markers. Triple-*MAPT*-mutant neurons also showed an elevated stress response signature linked to oxidative stress, ER stress and inflammatory stress with increased spontaneous cell death in culture (Garcia-Leon et al., 2018).

### Frontotemporal Lobar Degeneration With Tau Pathology Caused by *MAPT*^10 + 14^

This intronic mutation leads to tau pathology in both neurons and glial cells with increased production of 4R tau (Ghetti et al., 2015). In one published study, iPSCs were generated from a patient with the *MAPT* 10 + 14 mutation and isogenic control cells were derived using CRISPR/Cas9 technology (Imamura et al., 2016). As seen for neurons carrying the 10 + 16 mutation, 10 + 14 neurons exhibited an increased expression of 4R tau resulting in an increased 4R:3R tau ratio (Imamura et al., 2016). Notably, the authors describe accumulation of intracellular misfolded tau protein with formation of intracellular puncta and dots in 10 + 14 neurons. This misfolded tau was also released into the medium supernatant. 10 + 14 neurons presented with increased calcium levels after electrical stimulation and showed increased spontaneous cell death, which could be prevented by blockage of calcium influx via application of the AMPA receptor inhibitor CNQX or the NMDA receptor inhibitor AP-5 (Imamura et al., 2016), as similarly seen in 10 + 16 neurons. These findings highlight that similar mechanisms may contribute to increased cell vulnerability in patient neurons with different intronic *MAPT* mutations.

### Frontotemporal Lobar Degeneration With Tau Pathology Caused by *MAPT*^*V*337*M*^

The V337M *MAPT* mutation is located in exon 12 and is a missense mutation (Spillantini et al., 1996; Kar et al., 2005; Spina et al., 2017). It leads to increased phosphorylation and aggregation of tau and to an impaired binding of tau to microtubules (Alonso Adel et al., 2004; Kar et al., 2005). The V337M mutation causes neuronal, but no glial tau pathology and does not alter the ratio of 3R and 4R tau isoforms in contrast to the aforementioned *MAPT* mutations in exon 10 and intron 10.

In line with this finding, V337M patient iPSC-derived neurons did not aberrantly express 4R tau isoforms and an altered 4R:3R tau ratio was not seen (Ehrlich et al., 2015). However, and similar to N279K patient neurons, V337M neurons demonstrated an increased fragmentation of tau protein and contained an increased number of p-tau-positive neurons. In addition, V337M mutant neurons had an impairment of neurite outgrowth and were much more susceptible to rotenone-induced oxidative stress, which was partially reversable through GSK-3 inhibition and through the application of coenzyme Q10 (Ehrlich et al., 2015). Interestingly, MAGEH1 was also identified in V337M patient neurons as a neuroprotective molecule, similar to N279K neurons, further supporting its beneficial role in FTLD-tau (Ehrlich et al., 2015).

Induced pluripotent stem cell-derived neurons with the V337M mutation also demonstrated structural and functional alterations of the axon initial segment (AIS), which represents an area within neurons with unique cytoskeletal organization that contains tau-binding molecules and that initiates action potentials (Sohn et al., 2019). In this study, V337M neurons were either derived from FTLD-tau patients with the V337M mutation or by the introduction of the V337M mutation into human iPSCs via CRISPR/Cas9 technology. V337M mutant neurons showed shortening of the AIS and neuronal dysfunction with increased neuronal excitability after depolarization that was directly linked to an increased amount of ‘end-binding protein 3’ (EB3), one of the tau-binding molecules within the AIS. In fact, these alterations were normalized by reducing the levels of EB3 via siRNA, which resulted in elongation of the AIS and in the repair of AIS plasticity, as evidenced by lack of an abnormally increased firing rate upon depolarization (Sohn et al., 2019). Of note, these phenotypes were also rescued by repairing the V337M mutation in patient cells with CRISPR/Cas9 technology, demonstrating that structural and functional AIS pathology was directly driven by the V337M *MAPT* mutation (Sohn et al., 2019).

In a recent study, an isogenic organoid model of *MAPT* V337M-associated FTLD-tau was established to study disease-associated changes and mechanisms of early neural degeneration in a three-dimensional context using single cell RNA sequencing among other techniques (Bowles et al., 2021). Patient and gene-corrected iPSC-derived organoids were followed over 6 months of differentiation demonstrating accelerated differentiation of cortical glutamatergic neurons with earlier expression of glutamatergic signalling pathways and synaptic genes at 2 months and a significant reduction of excitatory deep and upper layer cortical neurons but not of interneurons at 4 and 6 months of culture of V337M organoids. These findings demonstrated selective excitatory neuron vulnerability in these organoids (Bowles et al., 2021). V337M organoids also showed a progressive upregulation and accumulation of total tau and p-tau, they demonstrated dysfunction of the early autophagy-lysosomal pathway, and they exhibited increased formation of stress granules. While abnormal splicing of *MAPT* was not observed, V337M organoids showed splicing dysregulation in genes associated with synaptic signaling and stress granule markers, further demonstrating dysfunctional synaptic maturation and increased stress responses in V337M organoids. Furthermore, V337M organoids presented with an accelerated and increased expression of the RNA-binding protein ELAVL4 that is known to regulate RNA splicing and that was found to bind *MAPT* RNA and to co-localize with the stress granules in V337M neurons. V337M organoids also demonstrated an increased susceptibility to glutamate-induced excitotoxicity, which could be rescued by the application of glutamate receptor inhibitors and by the PIKFYVE kinase inhibitor apilimod, providing a potential therapeutic target. Interestingly, neuronal disease phenotypes in V337M organoids were paralleled by an astrocyte-mediated neuroinflammatory response with an upregulation of interleukin-6, interleukin-8 and neuroinflammation signaling pathways in V337M astrocytes (Bowles et al., 2021). These findings highlight that iPSC-derived organoids represent an elegant platform to study neuronal but also glial pathology and, potentially, any glia-mediated mechanisms of neuronal degeneration over prolonged periods of time.

### Frontotemporal Lobar Degeneration With Tau Pathology Caused by *MAPT*^*R*406*W*^

This *MAPT* mutation is located in exon 13. Similar to the V337M mutation, it is a missense mutation that leads to increased phosphorylation, an impaired binding of tau to microtubules and increased aggregation of tau (Reed et al., 1997; Miyasaka et al., 2001; Alonso Adel et al., 2004; Kar et al., 2005). The pathology in the brains of patients is neuronal with formation of neurofibrillary tangles and with an unaltered 3R:4R tau isoform ratio (Ghetti et al., 2015). In addition, clinical and histopathological features of AD with formation of rare Aβ-positive neuritic plaques have been found in patients.

As described above for the *MAPT* 10 + 14 mutation, tau was misfolded in patient iPSC-derived *MAPT* R406W neurons. This misfolded tau protein was deposited within neurons as small puncta and dots and it was released into the supernatant of cultured neurons. Pathologic changes in *MAPT* R406W neurons were also linked to disturbed calcium homeostasis, as these neurons exhibited increased calcium levels after electrical stimulation and demonstrated increased spontaneous cell death linked to elevated calcium levels. In fact, blockage of calcium influx by aforementioned glutamate receptor inhibitors CNQX or AP-5 could prevent cell death in *MAPT* R406W neuronal cultures (Imamura et al., 2016).

An integrative system biology approach was applied to characterize transcriptome profiles in iPSC-derived neurons from a carrier of the *MAPT* R406W mutation versus those in isogenic control neurons and in comparison to postmortem brain tissue from FTLD-tau patients carrying the same *MAPT* R406W mutation (Jiang et al., 2018). The authors found 61 genes which were equally up- or downregulated in both patient iPSC-derived neurons and postmortem insular cortex. Functional annotation of these genes revealed enrichment for pathways involving calcium-dependent pre-synaptic function and GABAergic signaling with downregulation of various GABAergic receptors in both *MAPT* R406W iPSC-derived neurons and brains. Interestingly, improved gene-set enrichment analysis for GWAS (*i*-GSEA4GWAS) revealed that these 61 overlapping genes were significantly enriched for FTD risk variants and that SNPs within these 61 genes were more significantly associated with FTD risk than expected by chance (Jiang et al., 2018). In addition, the authors found that differential gene expression signatures in *MAPT* R406W iPSC-derived neurons also had some overlap with gene expression profiles from postmortem brain tissue of patients with PSP, a related disorder and another form of FTLD-tau, but not with gene expression signatures from postmortem brain tissue of patients with AD or FLD-TDP (Jiang et al., 2018).

Disease phenotypes in R406W neurons were also studied in neurons isolated from cerebral organoids. For this study, the authors dissociated cerebral organoids from R406W iPSCs and isogenic controls 30 days after initial differentiation and replated the neurons for analysis after an additional 30 days (Nakamura et al., 2019). In contrast to reports on neurons carrying other *MAPT* mutations, the R406W mutation led to reduced phosphorylation of tau in patient neurons with especially poor phosphorylation by GSK3β. Furthermore, patient R406W neurons exhibited increased fragmentation of tau protein with a particular increase in the amount of N-terminal tau fragments. Blocking experiments revealed that this increased fragmentation was driven by calpain cleavage. Tau was also mislocalized from axons to dendrites in patient neurons. In addition, axonal dystrophy with formation of βIII-tubulin-positive puncta and an impaired axonal transport of mitochondria along microtubles were seen in patient neurons. Both of these phenotypes could be rescued by adding the microtubule-stabilizing compound Epothilone D to the media, indicating disturbed but reversible microtubule function in R406W neurons (Nakamura et al., 2019).

### Frontotemporal Lobar Degeneration With Tau Pathology Associated With *MAPT*^*A*152*T*^

Although its role is still under debate, the *MAPT* A152T variant significantly increases the risk for FTD and AD and it has been described in rare patients with PSP or CBD (Coppola et al., 2012; Kara et al., 2012; Biswas et al., 2016). This *MAPT* variant is located in exon 7 and it can cause tau pathologic changes with an increase of soluble tau oligomers and a decrease of binding of tau to microtubules (Coppola et al., 2012).

The first iPSCs with the A152T variant were derived by Fong et al. (2013), who also generated isogenic control iPSCs as well as homozygous, bi-allelic mutant iPSCs via ZFN-mediated gene-editing technology. Cultures composed of A152T neurons demonstrated an increased vulnerability of especially dopaminergic neurons as similarly seen in PSP, which can be caused, though rarely, by *MAPT* A152T (Coppola et al., 2012; Fong et al., 2013). Differentiated A152T neurons also showed pronounced tau pathology, which was further aggravated in neurons homozygous for the variant. Both heterozygous and homozygous A152T neurons exhibited shortening, bending and fragmentation of neurites with a punctate staining pattern of tau. Furthermore, mislocalization of tau to the somatodendritic compartment was observed in neurons that were homozygous for the A152T variant. A152T neuronal cultures showed increased p-tau expression and increased numbers of p-tau-positive neurons. Western blot studies revealed increased proteolysis of tau, which was in part mediated by caspase cleavage. Interestingly, several of these tau pathologic changes were also observed in postmortem brain tissue from a PSP patient carrying *MAPT* A152T (Fong et al., 2013).

An increased 4R:3R tau isoform ratio was described in A152T patient neuronal cultures (Biswas et al., 2016). In this study, A152T neurons presented with an increased vulnerability under normal culture conditions and in the presence of the mTOR inhibitor rapamycin, as similarly seen for neurons with the 10 + 16 mutation. Likewise, increased rapamycin-induced vulnerability in A152T neurons was, at least partially, reversed by blockage of MMP-9 and MMP-2, which were also significantly upregulated and activated in A152T iPSC-derived neurons (Biswas et al., 2016). This study demonstrated that MMP-9 activation in A152T neurons is dependent on ERK phosphorylation, since inhibition of the ERK pathway decreased MMP-9 expression in these cells. In addition, ectopic expression of 4R tau with the A152T variant activated the ERK pathway to increase MMP-9 expression in HEK293 cells. Taken together, these observations suggested an upstream role of 4R tau on ERK- and MMP-9-mediated vulnerability in patient-derived neurons carrying the A152T variant in *MAPT* (Biswas et al., 2016).

In an independent study, A152T neurons displayed an accelerated and increased expression of tau with unaltered 4R:3R isoform ratio and of p-tau leading to p-tau accumulation (Silva et al., 2016). While aggregation of tau and tangle formation was not seen, the tau protein in A152T neurons showed reduced solubility and somatodendritic redistribution. Mass spectroscopy confirmed increased posttranslational modification of tau and provided, for the first time, a quantification of allele-specific expression of tau protein in human iPSC-derived neurons. Here, A152T neurons expressed relatively higher levels of A152T tau (about 60%) than non-mutant tau (about 40%) and this ratio remained constant throughout prolonged differentiation. A152T neurons also demonstrated protein expression profiles linked to activated autophagy, increased protein ubiquitination and elevated ER stress and showed an increased vulnerability toward oxidative stress, glutamate-mediated excitotoxicity as well as proteasomal and Aβ-(1–42)-induced stress. Notably, these stress responses could be rescued by reducing or disrupting the expression tau in these neurons (Silva et al., 2016). Also, Aβ(1–42)-mediated stress could also almost completely be prevented by the application of aforementioned tau degrader QC-01–175, as similarly seen in P301L neurons, resulting in more than 70% clearance of tau and p-tau in A152T neurons via proteasomal degradation (Silva et al., 2019).

The same group later applied a high-content imaging assay on A152T and control cortical neurons to screen for kinases and small molecules to reduce tau and p-tau expression as well as their somatodendritic redistribution (Cheng et al., 2021). This study revealed that the GSK-3 inhibitor CHIR-99021 and the kinase inhibitors enzastaurin and ruboxistaurin reduced p-tau in cell processes and cell body in A152T and control neurons and that the small molecule kinase inhibitors AT7519 and CGP-60474, as part of a collection of 44 kinase inhibitors, had similar effects in a screen on A152T neurons. Furthermore, the application of CHIR-99021 and enzastaurin resulted not only in reduced phosphorylation of tau but also in a decrease of total tau in these cells suggesting clearance of tau by these compounds and a potentially beneficial function of kinase inhibitors in patient neurons (Cheng et al., 2021).

### Additional Induced Pluripotent Stem Cell Models of Frontotemporal Lobar Degeneration With Tau Pathology Including Progressive Supranuclear Palsy and Corticobasal Degeneration

Several groups have published resource papers that report on the generation of iPSCs from patients with FTLD-tau without providing details on disease phenotypes in differentiated neural cells. The reports describe iPSC generation from a patient with the S305I *MAPT* mutation in exon 10 (Kovacs et al., 2008; Nimsanor et al., 2016a) and from a patient and a carrier with the R406W *MAPT* mutation in exon 13 (Rasmussen et al., 2016a, b) with generation of isogenic lines (Nimsanor et al., 2016b, c). A recent resource study of the TAU consortium reports on several iPSCs from patients with primary tauopathies, especially genetic forms of FTLD-tau, many of which are reviewed in this article (Karch et al., 2019). The authors also list iPSCs as resource from patients or carriers with the S305I (Kovacs et al., 2008), S305N (Hasegawa et al., 1999), and S305S (Stanford et al., 2000) *MAPT* mutations in exon 10, from patients or carriers with the G389R *MAPT* mutation in exon 13 (Murrell et al., 1999) as well as iPSCs from a CBD patient carrying the A152T mutation and from two PSP patients without *MAPT* mutations, providing additional opportunities for stem cell-based disease modeling. Table 5 provides a summary of alternative sources of FTLD-tau iPSCs.

**TABLE 5:** Additional sources of FTLD-tau iPSC lines.

Platform	Examples of iPSC lines	Link
TAU consortium	A152T, 10 + 16, P301L, S305I, S305N, S305S, V337M, G389R, R406W, R5H, PSP	https://www.neuralsci.org/tau/human-ipsc-lines
NINDS Human Cell and Data Repository	N279K, P301L, S305I, S305N, V337M, G389R, R406W	https://stemcells.nindsgenetics.org/
European Bank for Induced pluripotent Stem Cells (EBiSC)	P301L, P301S, 10 + 16, P301S/10 + 16	https://ebisc.org/

## Discussion

As reviewed in this article, iPSC technology allows to study FTLD-tau-associated changes in patient-derived neural cells. Most of the studies were performed on cortical neurons as the main affected cell type in FTLD-tau while few studies focused on other neural subtypes such as dopaminergic neurons, astrocytes or oligodendrocytes, which are also heavily involved by the disease. Main phenotypes included significant tau pathologic changes such as tau hyperphosphorylation, tau mis-splicing with increased and accelerated formation of 4R isoforms in mutations in exon 10 and intron 10, tau fragmentation, oligomerization and accumulation of misfolded tau as well mislocalization of tau to the somatodendritic compartment. They also encompassed disturbed microtubule function in patient neurons with impairment of neurite outgrowth, disturbed nucleocytoplasmic transport of proteins with formation of nuclear indentations as well as dysfunctional anterograde and retrograde transport of mitochondria along microtubules. Also, patient neurons presented with metabolic disarrangements with altered ATP production, an increased electrophysiological activity with formation of dysmorphic action potentials, significant calcium overload and increased glutamate-induced excitotoxicity, increased ROS production as well as pronounced vulnerability toward oxidative, Aβ-induced, proteasomal and ER stress.

Many of these disease phenotypes were reversable providing elegant platforms for high-throughput drug screening for compounds in pre-clinical settings to be able to potentially interfere at an early stage of the disease development process. In fact, human iPSC-derived drug screening platforms have been established (Wang et al., 2017; Cheng et al., 2021) and large numbers of patient-derived neural cells can readily be derived, cultured and tested in such dynamic cellular assays *in vitro*, in contrast to alternative approaches involving genetic mouse models or postmortem brain material from patients. Such drug screening assays may also involve the co-culture of patient iPSC-derived neurons with patient iPSC-derived glial cells such as astrocytes to test glia- or even neuron-mediated non-cell autonomous mechanism of disease development in neurodegenerative diseases, such as FTLD-tau. In this context, it should be noted that FTLD-tau patient iPSC-derived neurons, e.g., those carrying the 10 + 14 neurons or the R406W *MAPT* mutation, can secrete misfolded tau into the medium supernatant (Imamura et al., 2016) providing an opportunity study of tau seeding effects and mechanism of tau propagation in neuron-neuron or neuron-glia co-culture paradigms. Refinements of the reprogramming technology and application of genome editing strategies in patient-derived stem cells further improved iPSC-based disease modeling, such that ZFN, TALEN (transcription activator-like effector nucleases) and CRISPR/Cas9 technologies further reduced genetic heterogeneity and thus clonal variabilities for the differentiation of iPSCs into neural cell types of interest. These technologies have been applied in several of the FTLD-tau studies reviewed in this article (see also Tables 1–4).

While these findings highlight the importance of the iPSC technology in biomedical research, some limitations still have to be overcome. For instance, environmental factors are difficult to model and the overall number of patients in iPSC studies is generally low. Also, while the *in vitro*-differentiation of human iPSCs into neurons recapitulates neurogenesis *in vivo* with formation of all six tau isoforms over time, an extended differentiation is needed to express all tau isoforms *in vitro*, which is neither cost- nor time-efficient. As outlined here, many of the disease-associated changes in FTLD-tau neurons were already observed at early stages of differentiation with detection of similar pathologies in postmortem patient brain tissue in some of these studies. However, other typical disease-associated changes in patients’ brains such as the formation of p-tau-positive neurofibrillary tangles in neurons or p-tau-positive aggregates in glial cells have not been detected in human iPSC-derived cells. Thus, an acceleration of differentiation and an induction of premature aging may be necessary to shorten time of culture and to detect additional disease phenotypes in iPSC-derived neurons and glial cells. These approaches may involve the exposure to oxidative stress, introduction of DNA damage or impairment of DNA repair in differentiating cells (Miller et al., 2013), in part also addressing the limitation of lack of environmental cues. The culture of iPSC-derived cells as organoids (Seo et al., 2017; Bowles et al., 2021) and the transplantation of patient neural cells into rodent brains (Hargus et al., 2010) may provide additional alternatives since patient neurons could grow and communicate in a more physiological, three-dimensional context. The application of common immunohistochemical and biochemical tools as well as novel single cell technologies such as single cell proteomics or single cell RNA sequencing, as shown for *MAPT* V337M organoids (Bowles et al., 2021), may be very helpful in this context to detect previously unrecognized disease-associated changes. In addition, chimeric mice containing transplanted patient-derived neural cells could be used in pre-clinical settings to test for blood-brain-barrier-penetrance and *in vivo*-efficiency of compounds that had previously provided positive results in aforementioned high-throughput drug screening assays. Direct site-by-site comparisons of early versus aging-induced stages as well as comparisons with three-dimensional assays may be very helpful to further optimize iPSC-based disease modeling.

Induced pluripotent stem cell models of FTLD-tau with increased 4R tau expression, such as those linked to mutations in exon 10 and intron 10, require thorough assessment of 4R and 3R tau expression and their ratio in neural cells for model validation and to derive meaningful conclusions of translational value. While potentially challenging, given the predominant expression of fetal 3R tau in early iPSC-derived neurons, altered 4R:3R tau isoform ratios were reported in various stem cell models (see Tables 1–4 for details). It is possible that time frames of cell differentiation may have to be adjusted for certain differentiation protocols to assess the 4R:3R tau isoform ratio and to extract optimal results for model validation. Also, continual optimization of differentiation protocols for neuronal subtypes and glial cells as well as comparative analyses of phenotypes in different neural cell types, e.g., excitatory versus inhibitory cortical neurons or cortical versus subcortical/brainstem-type neurons and glial cells, are desirable to further strengthen iPSC-based modeling systems.

While disease phenotypes appeared to be robust in summarized studies, larger numbers of patient donors for iPSC generation would further support detection of smaller, yet critically important differences between groups. This is especially important when modeling sporadic forms of neurodegenerative diseases to account for donor- and aforementioned stem cell clone-related variabilities since isogenic control cells cannot be derived. In this context, the *MAPT* haplotype, either H1 or H2, of donors and stem cell lines could also play an important role since the H1 *MAPT* haplotype is associated with a higher risk for developing PSP, CBD, AD, and Parkinson’s disease (PD) (Kwok et al., 2004; Myers et al., 2005; Hoglinger et al., 2011; Kouri et al., 2015). Interestingly, allele-specific expression of *MAPT* transcripts with increased H1 *MAPT* transcripts has been reported in H1/H2 heterozygous iPSC-derived neurons from disease-free donors (Beevers et al., 2017). On the other hand, a comparative study on healthy-donor iPSC-derived neurons carrying either the H1/H1 or the H2/H2 *MAPT* haplotype did not reveal major haplotype-specific differences in tau expression but it showed significantly higher levels of conformationally altered MC1-positive insoluble tau in neurons carrying the H2/H2 haplotype, while neurons with the H1/H1 haplotype demonstrated an increased expression of alpha-synuclein that accumulates in neurons in PD (Strauss et al., 2021). Thus, genetic variants can modulate disease-associated processes, which is a very interesting aspect to explore in future iPSC-based studies on sporadic and familial forms of tauopathies including FTLD-tau.

Comparative studies with iPSCs from patients with other forms of FTD/FTLD such as FTLD-TDP may further identify mutual pathways of neurodegeneration such as pathways linked to neuroinflammation. Recent progress in iPSC research led to protocols that efficiently derive microglia from human iPSCs (Muffat et al., 2016; McQuade et al., 2018; Guttikonda et al., 2021). Thus, future studies could involve patient-derived microglia as pure cultures or as co-cultures with neurons and other glial cells as potential important modulators to study the role of neuroinflammation in FTLD-tau. Altogether, these various opportunities clearly highlight the strong value of iPSCs for research on neurodegenerative diseases including FTD/FTLD-tau.

## Author Contributions

RK, AM, PC, and GH contributed to the writing of this manuscript. All the authors contributed to the article and approved the submitted version.

## Conflict of Interest

The authors declare that the research was conducted in the absence of any commercial or financial relationships that could be construed as a potential conflict of interest.

## Publisher’s Note

All claims expressed in this article are solely those of the authors and do not necessarily represent those of their affiliated organizations, or those of the publisher, the editors and the reviewers. Any product that may be evaluated in this article, or claim that may be made by its manufacturer, is not guaranteed or endorsed by the publisher.
